# Sales for anti-angiogenic drugs

**DOI:** 10.18632/oncotarget.17373

**Published:** 2017-04-23

**Authors:** Domenico Ribatti

**Affiliations:** Department of Basic Medical Sciences, Neurosciences, and Sensory Organs, University of Bari Medical School, National Cancer Institute, Bari, Italy

**Keywords:** anti-angiogenesis, tumor growth

In 1971, Judah Folkman developed the concept of “anti-angiogenesis” as a potential treatment of cancer due to “the prevention of new vessel sprouts from penetrating into early tumor implant” [1]. Beginning in the 1980's, the industry exploited the field of anti-angiogenesis for creating new therapeutic molecules in angiogenesis-dependent diseases. In 2004, the first anti-angiogenic drug, bevacizumab was approved by the FDA for the management of advanced colon cancer. A number of anti-angiogenic agents have currently been approved for cancer treatment, alone or in combination with other anti-tumoral drugs. Moreover, angiogenesis inhibitor combined with chemotherapy could induce vessel normalization and chemotherapeutic drugs could be delivered to tumor and exert their effects [2]. The concept of “normalization” of tumor vasculature was introduced in 2001. Accordingly, VEGF/VEGFR therapies induce morpho-functional normalization of tumor blood vessels, favoring an increase in blood flow and release of cytotoxic drugs.

Despite impressive performances in animal models, however, inhibitors are not performing nearly as well in humans, spelling the end for several candidates and at least one company. Anti-angiogenic treatments lead to only a 3-6 months increase in progression free survival (PFS), followed by a relapse in tumor angiogenesis and growth.

While colorectal, lung and breast cancer patients have responded, pancreatic cancer patients have not shown survival advantages when treated with anti-angiogenic monotherapy or combinations of anti-angiogenic agents with chemotherapy. Moreover, responses to anti-angiogenic drugs vary between primary tumors and their metastases.

The FDA uses as a criterion to approve a new anti-cancer drug the improve in patient survival. Despite anti-angiogenic therapy has increased PFS of patients with cancer, the pooled results showed that overall survival improvement was very limited [3, 4]. The effective therapy is ineffective against cancer stem cells, which replenish the tumor, causing relapse. Moreover, hypoxia stimulates tumor angiogenesis, favoring tumor growth and metastasis [5–7].

The limitations of applying angiogenesis inhibitors are attributed to drug resistance, metastasis promotion and reduced delivery of chemotherapeutic agents, resulted from the dramatic decrease of tumor vasculature [8].

Multiple mechanisms of drug-induced resistance against angiogenesis inhibitors exist. Some anti-angiogenic molecules are more capable to induction of resistance than others and this event is responsible of the discontinuation of the therapy. Moreover, it is necessary to select drugs with a low resistance to prevent or overcome the development of resistance, and combine different molecules to improve the therapeutic effect [8].

Most of the FDA-approved drugs are able to inhibit a single angiogenic molecule, while tumor cells are able to synthesize multiple angiogenic molecules in different stages of their progression. Finally, it is also important to reach a general consensus as concerns the treatment timeline when anti-angiogenic drugs are used.

Since Napoleone Ferrara at Genentech singled out VEGF as the most important factor involved in the process of blood vessel formation in 1989 [9], a much broader understanding of the multiple pathways and receptors involved in angiogenesis has developed. Anti-angiogenesis therapy has grown since 2005 into a global market valued at approx. $12.0 billion (2010) and predicted to reach $18.0 billion by 2015. The market is dominated by various classes of drugs collectively known as anti-VEGFs, for both the ophthalmology and oncology use. The sales of anti-VEGFs drugs in oncology are exceeding 10 billion dollars per year, taking in account that bevacizumab alone accounting for almost 7 billion, making it presently the drug with the seventh highest revenues.

These enormous socioeconomic investments re-justified in the light of the limited efficacy of anti-angiogenic drugs, particularly in the context of tumor progression with the consequence that at the present state, profitability overrides health benefits [10]. The most important objective is to establish validated biomarkers with the aim to personalize VEGF inhibitors and select responding patient sub-populations [11].
